# Sensing a CO-Releasing Molecule (CORM) Does Not Equate to Sensing CO: The Case of DPHP and CORM-3

**DOI:** 10.1021/acs.analchem.3c01495

**Published:** 2023-06-01

**Authors:** Dongning Liu, Xiaoxiao Yang, Binghe Wang

**Affiliations:** Department of Chemistry and Center for Diagnostics and Therapeutics, Georgia State University, Atlanta, Georgia 30303, United States

## Abstract

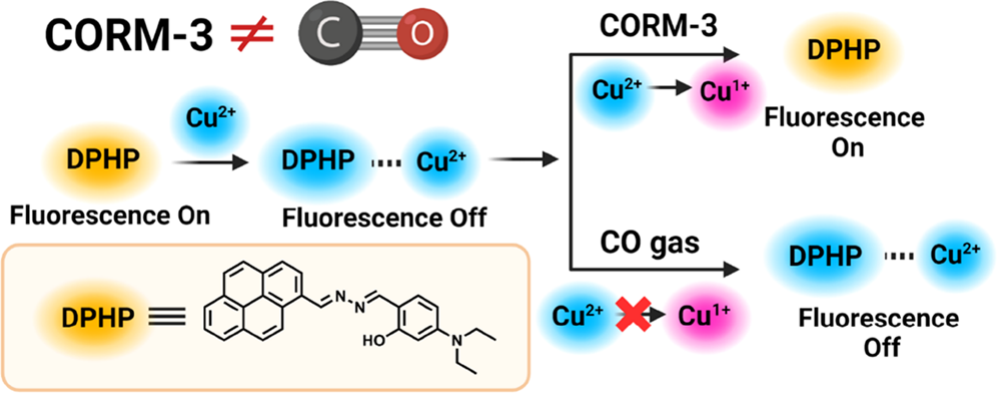

Carbon monoxide (CO) is an endogenous signaling molecule with demonstrated pharmacological effects. For studying CO biology, there is a need for sensitive and selective fluorescent probes for CO as research tools. In developing such probes, CO gas and/or commercially available metal-carbonyl-based “CO-releasing molecules” (CORMs) have been used as CO sources. However, new findings are steadily emerging that some of these commonly used CORMs do not release CO reliably in buffers commonly used for studying such CO probes and have very pronounced chemical reactivities of their own, which could lead to the erroneous identification of “CO probes” that merely detect the CORM used, not CO. This is especially true when the CO-sensing mechanism relies on chemistry that is not firmly established otherwise. Cu^2+^ can quench the fluorescence of an imine-based fluorophore, DPHP, presumably through complexation. The Cu^2+^-quenched fluorescence was restored through the addition of CORM-3, a Ru-based CORM. This approach was reported as a new “strategy for detecting carbon monoxide” with the proposed mechanism being dependent on CO reduction of Cu^2+^ to Cu^1+^ under near-physiological conditions (Anal. Chem.2022, 94, 11298−113063592608110.1021/acs.analchem.2c01948). The study only used CORM-3 as the source of CO. CORM-3 has been reported to have very pronounced redox reactivity and is known not to release CO in an aqueous solution unless in the presence of a strong nucleophile. To assess whether the fluorescent response of the DPHP-Cu(II) cocktail to CORM-3 was truly through detecting CO, we report experiments using both pure CO and CORM-3. We confirm the reported DPHP-Cu(II) response to CORM-3 but not pure CO gas. Further, we did not observe the stated selectivity of DPHP for CO over sulfide species. Along this line, we also found that a reducing agent such as ascorbate was able to induce the same fluorescent turn-on as CORM-3 did. As such, the DPHP-Cu(II) system is not a CO probe and cannot be used to study CO biology. Corollary to this finding, it is critical that future work in developing CO probes uses more than a chemically reactive “CO donor” as the CO source. Especially important will be to confirm the ability of the “CO probe” to detect CO using pure CO gas or another source of CO.

## Introduction

With the establishment of the endogenous production of carbon monoxide (CO) through heme degradation by heme oxygenases^1−3^ and the validation of its various pharmacological functions,^4,5^ there comes the need for developing fluorescent probes and/or sensors for CO for highly sensitive and highly selective detection in solution, in cell culture, and tissues.^6^ Along this line, Chang and colleagues pioneered an innovative approach by cleverly taking advantage of Pd-mediated carbonylation chemistry for CO sensing.^7^ In parallel, He and colleagues developed the first protein-based CO sensing method by taking advantage of the conformational change of a hemoprotein upon binding to CO.^8^ Subsequently, there are other innovative approaches reported, including the application of Pd-mediated de-allylation reaction for fluorophore activation and thus CO detection.^9−12^ Recently, we reported a strategy of designing CO probes through *de novo* construction of a fluorophore via Pd-mediated carbonylation chemistry, leading to exclusivity and very high sensitivity for CO detection and quantification.^13^

In developing fluorescent CO probes, typically used CO sources include CO gas, commercially available “CO-releasing molecules” (CORMs) such as CORM-2 and CORM-3,^14,15^ or metal-free CO prodrugs.^16^ Ideally, the source of CO should not make any difference in probe sensitivity and a valid probe should be able to detect CO regardless of its source. After all, CO is CO. However, recent revelations of the lack of reliable CO production by some CORMs^15,17−21^ and their pronounced chemical reactivity^19,22−34^ have raised the question of whether one can rely on results by only using a CORM as the sole CO source in fluorescent probe development for CO.^6^ This is especially true when the “CO sensing” mechanism is proposed to be based on chemistry, which is either unlikely to happen or otherwise has no precedents. For example, there were reports of CO probes relying on the ability of CO to reduce an aromatic nitro group (to an amine) as a way to sense CO. Such a reaction would be unprecedented in organic chemistry under near-physiological conditions. Incidentally, the evaluation of such “CO probes” only used some redox-active CORMs such as CORM-2 and CORM-3 as the sole “CO source” without corroboration by using CO gas. Later, such “CO probes” were found not to sense CO, but merely the metal-carbonyl complex used for assessing these probes.^6,27,35,36^ As a result, such probes cannot be used for studying CO biology. It should be mentioned that there have been recent reports of fluorescent probes for Ru-based CORMs. These probes depend on the chemical reactivity of the CORM itself, not CO, to reduce an aryl nitro group^35−38^ and to remove an allyl group.^39^ However, these publications specify their intended use being for detecting a CORM, not CO. With all of these in mind, there is an urgent need to ascertain the ability of a “CO probe” to truly sense CO but not the CO donor itself before its application in studying CO biology.

**Figure 1:**
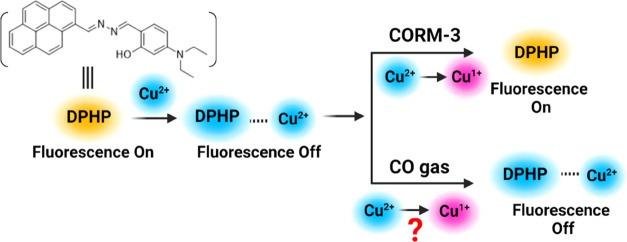
Originally proposed mechanism for DPHP to sense CORM-3 and CO gas (not).

Recently, a Cu^2+^-assisted CO probe (DPHP, Figure 1) was reported as a “strategy for detecting carbon monoxide,” with utility in CO bioimaging applications.^40^ Sensing mechanism was proposed to go through Cu^2+^-mediated fluorescence quenching of DPHP and CO reduction of Cu^2+^ to Cu^1+^, leading to the fluorescence recovery of DPHP (Figure 1). Specifically, Cu^2+^ was said to complex with DPHP with the imine and hydroxy groups, leading to fluorescence quenching of DPHP. Upon exposure to CO introduced by CORM-3, Cu^2+^ was said to be reduced by CO, leading to the removal of the complexed Cu^2+^ and thus fluorescence recovery/turn-on. For several reasons, we were intrigued by the proposed sensing mechanism and the stated ability of this probe to sense CO in solution and *in vivo*. First, despite its name as a “CO-releasing molecule,” CORM-3 is known NOT to release CO in an aqueous solution unless in the presence of a strong nucleophile such as a thiol or a sulfite species.^19,20^ Second, CORM-3 is a known reducing agent.^24−28^ We wonder how to deconvolute the reductive effects of CO and CORM-3 in this proposed sensing mechanism. Third, there is no known report of CO’s ability to reduce Cu^2+^ to Cu^1+^ in an aqueous solution at ambient temperature,^41,42^ to the best of our knowledge. All the reported CO reduction of Cu^2+^ to Cu^1+^ was conducted under forcing conditions at an elevated temperature.^41,42^ Thus, we wonder why the DPHP complex is so special. Fourth, Cu^1+^ has been reported to be easily air-oxidized to Cu^2+^ with a second-order rate constant of 3.1 × 10^4^ M^–1^ s^–1^.^43^ The competition between CO and O_2_ seems to heavily favor the oxidation direction. This is especially true considering the similar water solubility of O_2_ (1.25 mM) and CO (1 mM) and the fact there is about 20% O_2_ in the air and little CO. The concentration ratio between O_2_ and CO is estimated to be about 15000:1 in solution at the stated CO detection limit (17 nM) for the DPHP-Cu(II) system. It is true that chelation may change the redox chemistry of Cu^2+^ and Cu^1+^. Nevertheless, it is an issue worth assessing. Fifth, in the original publication, DPHP was shown to have three coordination bonds with Cu^2+^ (Scheme S1) involving two adjacent nitrogen atoms, which would afford a geometry inconsistent with the widely known Cu^2+^ geometry.^44,45^ Sixth, DPHP was said to be selective in detecting CO over sulfide. Given the known reactivity of sulfide with Cu^2+^ and the insoluble nature of CuS, one would expect sulfide at an adequate concentration to scavenge Cu^2+^ and thus turn on the fluorescence of the DPHP-Cu(II) complex. Therefore, we were puzzled by this selectivity and became interested in examining whether the fluorescent turn-on response of the DPHP-Cu(II) mixture to CORM-3 was truly due to CO, a prerequisite for DPHP to be called a CO probe, and whether the probe has the stated selectivity. For all of these, we have synthesized this probe, conducted a thorough assessment, and came to the conclusion that this probe does not detect CO and can detect sulfide, CORM-3, or ascorbate because of their respective ability to precipitate Cu^2+^ or reduce Cu^2+^ to Cu^1+^. Therefore, the DPHP-Cu(II) complex is not a “CO probe” and can respond to many species including redox-active metal complexes, ascorbate, and sulfide. Below, we describe our detailed study.

## Experimental Section

### Material and Instruments

Chemical reagents were purchased from Sigma-Aldrich (Saint Louis, MO) and/or Oakwood (Estill, SC). Solvents were purchased from Fisher Scientific (Pittsburgh, PA), and dry solvents were prepared by a Vigor Tech purification system (Houston, TX). Certificated pure CO calibration gas was purchased from GASCO (Oldsmar, FL). UV–vis absorption spectra were obtained by using a Shimadzu PharmaSpec UV-1700 UV–visible spectrophotometer (Kyoto, Japan). Fluorescence spectra were recorded on a Shimadzu RF5301PC fluorometer (Kyoto, Japan). ^1^H NMR (400 MHz) and ^13^C NMR (101 MHz) were acquired with a Bruker AV-400 MHz Ultra Shield NMR. Crystal data were acquired by the Emory Crystallography Center.

### Synthesis of the DPHP

DPHP was synthesized following a literature procedure.^40^ The detailed procedures and compound characterizations are described in the Supporting Information.

### Experimental Procedures for the Spectroscopic Work

The DPHP stock solution was prepared in CH_3_CN at a concentration of 1 mM. As an example, 0.19 mg of DPHP was weighed by microbalance and 461 μL of CH_3_CN was added to get 1 mM DPHP solution. All DPHP solution for spectroscopic experiments was prepared in a CH_3_CN/H_2_O solution (v/v = 1:1) with a final concentration of 10 μM. As an example, 750 μL of deionized water, 735 μL of CH_3_CN, and 15 μL of DPHP stock solution (1 mM) were added to a 1.5 mL cuvette to get a 10 μM DPHP solution. DPHP-Cu(II) refers to the Cu^2+^-quenched DPHP (10 μM each) solution.

### Spectroscopic Experiments of DPHP

The UV and fluorescence experiments of DPHP (10 μM, CH_3_CN/H_2_O solution (v/v = 1:1)) were carried out at room temperature. The fluorometer instrument parameters were set as λ_ex_ = 430 nm, 3.0 nm excitation bandwidth, 3.0 nm emission bandwidth, and high sensitivity of detection. All experiments were done in triplicate.

### Effects of CORM-3 on the Fluorescence of DPHP

CORM-3 and Cu^2+^ were prepared as 10 mM stock solutions. As an example, 0.59 mg of CORM-3 was weighed by a microbalance, and 201 μL of deionized water was added to get a 10 mM CORM-3 stock solution. For the Cu^2+^ quenching experiments, after preparing 10 μM DPHP solution in 1.5 mL cuvettes, 1.5 μL (1 equiv), 15 μL (10 equiv), and 30 μL (20 equiv) of Cu^2+^ stock solution (10 mM) were added to the cuvettes separately and mixed by pipetting and releasing. The spectrum was recorded for 180 s at 37 °C. For the effects of CORM-3 on DPHP, 1.5 μL (1 equiv), 15 μL (10 equiv), and 30 μL (20 equiv) of CORM-3 stock solution (10 mM) were added to the DPHP-Cu(II) solutions (10 μM each) separately and mixed by pipetting and releasing. The spectrum was recorded for 180 s at 37 °C. For the experiments using an extended period, the fluorescence intensity was recorded at the 1-, 2-, and 20-h time points at 37 °C. The cuvette images were taken under UV light (365 nm) (Figure 4a,b).

### Effects of CO Gas on the Fluorescence of DPHP

For the effects of CO gas on the fluorescence of DPHP, a rubber stopper was used to seal the 3 mL cuvette, which has 2 mL of DPHP-Cu(II) solutions (10 μM each). Then, 1 mL of air in the cuvette was removed by a syringe and 1 mL of pure CO gas was injected into the cuvette. After rigorous mixing (shaking), the data were recorded every 20 s at 37 °C. For Figure 5B, CO gas was directly bubbling into the DPHP-Cu(II) solution (both 10 μM) for 1 min, then the data were recorded at 37 °C.

### Effects of Sodium Ascorbate on the Fluorescence of DPHP

Sodium ascorbate was prepared as a 10 mM stock solution. As an example, 0.80 mg of sodium ascorbate was weighed by microbalance, and 405 μL of deionized water was added to get a 10 mM stock solution. For the effects of sodium ascorbate on DPHP, 1.5 μL (1 equiv), 15 μL (10 equiv), and 30 μL (20 equiv) of sodium ascorbate stock solution (10 mM) were added to the DPHP-Cu(II) solutions (both 10 μM) separately and mixed by pipetting and releasing. The spectrum was recorded for 180 s at 37 °C.

### Effects of Thiol Species on the Fluorescence of DPHP

Thiol species were prepared as 10 mM stock solution. For the effects of thiol species on the fluorescence of DPHP, 1.5 μL (1 equiv) of thiol species stock solution (10 mM) was added to the DPHP-Cu(II) solutions (10 μM each) and mixed by pipetting and releasing. After the reading became stable, 1.5 μL (1 equiv) of Cu^2+^ stock solution was added and mixed by pipetting and releasing. These steps were repeated another 2 times to complete this experiment.

## Result and Discussion

### Synthesis and Structural Confirmation of DPHP

As the first step of the validation work, we synthesized DPHP following the literature procedure.^40^ Routine NMR and MS work as well as X-ray crystallographic work were used to ensure the identity of DPHP (Figures S4–S9 and Table S1). Indeed, all of the characterization data support the structure of DPHP as stated.

### Confirmation of Literature Findings

**Figure 2:**
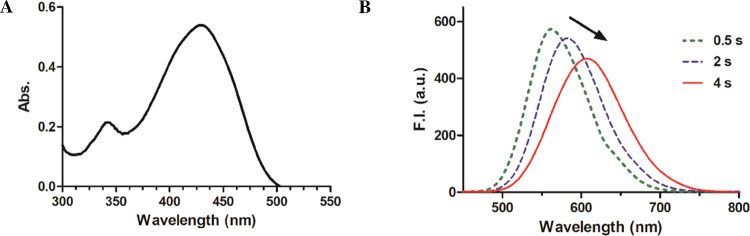
Spectroscopic properties of DPHP (10 μM) in CH_3_CN/H_2_O solution (v/v = 1:1). (A) UV spectrum of DPHP. (B) Fluorescence spectra of DPHP were tested with different response time settings. (λ_ex_ = 430 nm, bandwidth = 3 nm).

Before we assessed the probe’s ability to sense CO using a pure CO source or a CORM, we were interested in confirming literature findings of the probe’s spectroscopic properties and its response to the same CORM as described in the original publication. We first checked the spectroscopic properties of DPHP. As shown in Figure 2A, the UV–vis spectrum of DPHP in CH_3_CN/H_2_O (v/v = 1:1, λ_max_ = 430 nm) is in excellent agreement with that of the original publication. Such results serve as secondary validation of the literature findings in this regard. At first glance, the corresponding fluorescence spectra also seemed like a good match with that of the literature report (Figure 2B, λ_ex_ = 430 nm). However, we did observe what seemed like photostability issues. When the fluorescence spectra were acquired by using different settings for response time, we observed time-dependent shifts in both the λ_em_ and emission intensity. Within the window of the response time of 0.5–4 s, the λ_em_ of DPHP shifted from 555 to 609 nm, accompanied with decreased fluorescence intensity. This spectroscopic shift represents a new finding. For subsequent spectroscopic studies, we chose a response time of 2 s because this setting led to a λ_em_ of 582 nm, which is similar to what was reported in the original publication (λ_em_ = 590 nm). We did not pursue the mechanistic reasons for the observed spectroscopic shifts when different response time was used because it is not directly related to the theme of this study. Nevertheless, such shifts suggest that the photochemical stability issues of the probe need to be taken into consideration for data reproducibility assessments.

**Figure 3:**
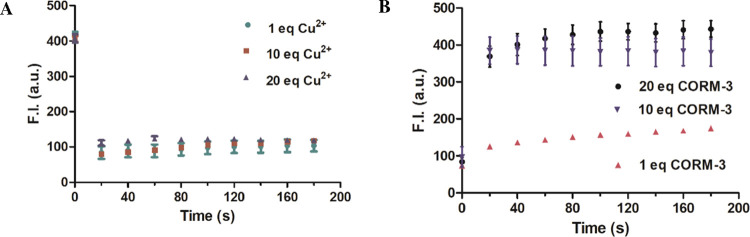
Time-dependent fluorescent changes of DPHP solution. (A) Effects of Cu^2+^ (1, 10, and 20 equiv) on the fluorescence of DPHP solution (10 μM). (B) Effects of CORM-3 (1, 10, and 20 equiv) on the fluorescence of DPHP-Cu(II) solution (10 μM each) (*n* = 3, mean ± SD, λ_ex_ = 430 nm, λ_em_ = 582 nm, bandwidth = 3 nm).

**Figure 4:**
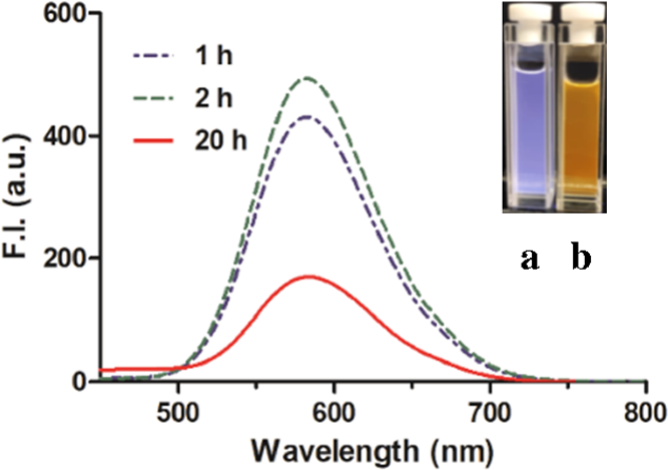
Fluorescent changes after CORM-3 (20 equiv) addition in the DPHP-Cu(II) solution (10 μM). (a) Visual image of the DPHP-Cu(II) solution after CORM-3 addition (20 h) (365 nm). (b) DPHP only (365 nm) (λ_ex_ = 430 nm, bandwidth = 3 nm).

Next, we examined the spectroscopic responses of DPHP to Cu^2+^. As shown in the original publication and Figure 1, Cu^2+^ was proposed to interact with DPHP, leading to fluorescence quenching. By adding 1 or 20 equiv Cu^2+^ to 10 μM DPHP solution (CH_3_CN/H_2_O, v/v = 1:1), fluorescence was quenched to 20% of the original level within 20 s (Figure 3A), which is in agreement with the findings in the original publication. Furthermore, we examined the spectroscopic responses of the DPHP-Cu(II) solution to CORM-3. As described in the original publication, the addition of CORM-3 at 1, 10, or 20 equiv (Figure 3B) led to fluorescence intensity increases of the DPHP-Cu(II) solution (10 μM, each) within 20 s. Such results are similar to that of the original publication and show that the fluorescence of the DPHP-Cu(II) solution is capable of detecting CORM-3. Overall, the results described above are generally consistent with literature findings in terms of the probe’s spectroscopic properties, fluorescence quenching by Cu^2+^, and the ability to detect CORM-3 by the DPHP-Cu(II) complex, except for the implication of the stated LOD of 17 nM due to the relatively weak response to CORM-3 at 1 equiv shown in the aforementioned study. Further, the *K*_d_ value for the DPHP-Cu(II) complex was not determined in the original publication and whether the reaction kinetics are such that detection at a low nM range is feasible. Both would impact the detection limit and show nonlinearity. Moreover, in conducting all of the experiments with CORM-3, we also observed photostability issues with the DPHP-Cu(II)-CORM-3 mixture. Specifically, after studying the spectroscopic properties of this mixture for 180 s (Figure 3), we also studied the photostability for an extended period of time. As shown in Figure 4, the spectroscopic properties of the DPHP-Cu(II)-CORM-3 solution were stable within 2 h but showed substantial changes at the 20-h point. For example, after incubation at 37 °C overnight, the fluorescence intensity of the mixture decreased to around 40% of the original level. The visual appearance of the solution under UV at 365 nm also changed from yellow to blue after 20-h incubation (Figure 4a,b). Such results suggest chemical and/or photochemical stability issues of the fluorescent complex. We did not study the chemical identity of the different species and instead just focused on examining the validity of using such a system for sensing CO.

### New Findings about This Probe Using CO Gas

**Figure 5:**
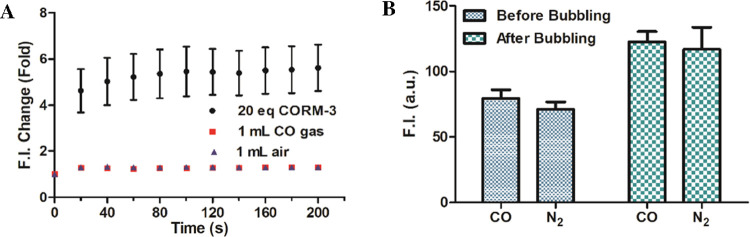
Effects of CO gas on the fluorescence of DPHP-Cu(II) solution (10 μM each). (A) Effects on the fluorescence (in comparison with the F.I. at 0 s) of the DPHP-Cu(II) solution (10 μM each) by CO gas addition (1 mL) (20 equiv CORM-3 and 1 mL air were used as controls). (B) Effects on the fluorescence of the DPHP-Cu(II) (10 μM each) solution by bubbling CO gas for 1 min (nitrogen gas was used as a control) (*n* = 3, mean ± SD, λ_ex_ = 430 nm, λ_em_ = 582 nm, bandwidth = 3 nm).

After confirming the spectroscopic properties of DPHP under various conditions and in response to Cu^2+^ and CORM-3 as described in the original publication, we were certain of two things. First, the probe that we used in the study was the same one as reported in the literature. Second, the results related to CORM-3 and DPHP-Cu(II) published in the original paper were of high quality and reproducibility. However, we do not interpret such results as indications that DPHP was shown to sense CO and this copper-mediated fluorescence response of DPHP to CORM-3 represents a new “Strategy for Detecting Carbon Monoxide,” as stated by the original publication. We raise this issue based on two rationales. First, CORM-3 is chemically reactive and is a far stronger reducing agent than CO at ambient temperature.^6,25^ There have already been similar cases reported,^27,35−39^ in which the stated “fluorescent probes for CO” turned out to only sense the CORM used as the CO source, but not CO itself. As such, there is a consensus in the field that the development of CO probes needs to use an unambiguous source of CO for assessing the “fluorescent CO probes.”^6^ In this particular publication, only the highly reactive CORM-3 was used as the CO source, leaving open many possibilities. Second, the proposed mechanism for this fluorescent probe to sense CO is not consistent with known chemistry. This in a way is similar to the aryl-nitro-based “CO probes,” which relied on “CO reduction” of such an aryl nitro group as the mechanism of sensing and yet such chemistry had/has no precedent and is not in agreement with known CO chemistry.^27^ For all of these reasons, we were interested in assessing the ability of DPHP to sense CO from an unambiguous CO source. Specifically, we used CO gas to assess the sensing ability of DPHP. As shown in the original publication and our results described in Figure 3B, the addition of 20 equiv of CORM-3 led to the fluorescent recovery of the DPHP-Cu(II) solution (10 μM each) within 20 s. In this case, we replaced the 20 equiv of CORM-3 (200 μM) with 1 mL of pure CO gas (the total volume of the cuvette is 3 mL). Because CO solubility in water is around 1 mM, the amount of CO available is at least 5-fold higher than using 20 equiv of CORM-3 (200 μM) assuming that CORM-3 would release a stoichiometric amount of CO (it does not!). As shown in Figure 5A, the CO gas group did not lead to meaningful fluorescence recovery of the DPHP-Cu(II) solution compared with the air treatment group as a control. In contrast, the addition of CORM-3 led to a 5-fold increase in the fluorescence intensity of the DPHP-Cu(II) solution. As such, CO gas did not play the same role as CORM-3.

After the experiments showed a lack of response of DPHP-Cu(II) solution to CO gas, we did further confirmation work by bubbling excessive amounts of CO for 1 min. As shown in Figure 5B, the fluorescence intensity of both the CO gas group and the N_2_ control group increased after bubbling the DPHP-Cu(II) solution for 1 min. The reason might be attributed to the stability issue of the DPHP-Cu(II) system, as we did observe a slow increase of fluorescence signal of the newly prepared DPHP-Cu(II) system on its own, which could be accelerated by bubbling a gas. Nevertheless, there is no significant difference between the CO and N_2_ treatment groups, confirming the inability of DPHP-Cu(II) to respond to CO.

### If CO Does Not Turn On the Fluorescence, What Is the Reason for the Fluorescence Turning On?

**Figure 6:**
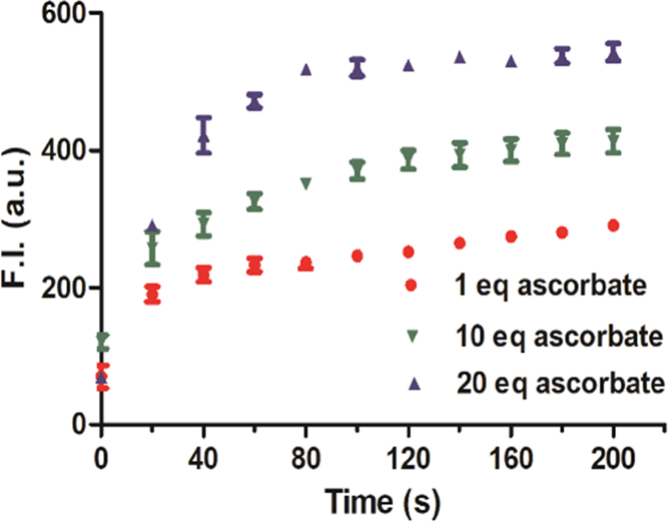
Effects of ascorbate (1, 10, and 20 equiv) on the fluorescence of DPHP-Cu(II) solution (10 μM each). Sodium ascorbate was used in the experiments (*n* = 3, mean ± SD, λ_ex_ = 430 nm, λ_em_ = 582 nm, bandwidth = 3 nm).

As CO itself has been proven not to turn on fluorescence by the originally proposed reduction of Cu^2+^ of the DPHP-Cu(II) solution, it is reasonable to deduce that the redox activities of CORM-3 could be the reason for such reduction reaction. To probe this issue, we sought to examine the effect of an agent known to reduce Cu^2+^ to Cu^1+^. Specifically, sodium ascorbate is widely used in the CuAAC reaction (Cu(I)-catalyzed azide-alkyne cycloaddition) to keep copper in the Cu^1+^ state.^46^ As expected, the addition of sodium ascorbate led to fluorescence turn-on of the DPHP-Cu(II) solution in a concentration-dependent manner (Figure 6). Such effects are similar to that of CORM-3 (Figure 3B). It is also important to note that the fluorescence level remained high for a period longer than what we expected, if air oxidation of Cu^1+^ had a second-order rate constant of 3.1 × 10^4^ M^–1^ s^–1^ under the current experimental conditions.^43^ The observed results mean that Cu^1+^ is not oxidized back to Cu^2+^ at a sufficiently high level to quench DPHP within the time frame of the experiments. To probe this issue, we first tested DPHP solution responses to Cu^1+^ and found a lack of fluorescence response to the addition of Cu^1+^ (Figure S1). Such results are consistent with what was reported in the original publication. Second, we also bubbled the 10 equiv sodium ascorbate group with oxygen for 5 min to see whether Cu^1+^ would be oxidized to Cu^2+^ and quench DPHP fluorescence again. As shown in Figure S2, the “DPHP-Cu(II)” solution remained in the turn-on state, which means Cu^1+^ was not converted back to Cu^2+^ quickly. Such results were initially puzzling to us because of the known instability of Cu^1+^ in the solution. Because in an air-saturated solution, free Cu^1+^ is known to be oxidized to Cu^2+^ within 25 ms.^47^ Without oxygen and at a lower pH, Cu^1+^ is known to disproportionate into Cu^2+^ and Cu^0^ (s); in neutral buffer, Cu^1+^ has been reported to form the cuprous oxide (Cu_2_O) as precipitation; in basic solution, Cu^1+^ forms the metastable CuOH and gradually decomposes to the sparingly soluble Cu_2_O form.^47^ The pH of the Cu^2+^-quenched DPHP solution was measured to be about 8. Under such conditions, Cu^1+^ is expected to form Cu_2_O or CuOH. Since neither would lead to Cu^2+^ formation, this might be the reason that Cu^1+^ did not go through air oxidation and then quench the fluorescence of the DPHP solution. In the end, it seems that the reducing ability of CORM-3 is likely the reason for its ability to turn on the fluorescence of the DPHP-Cu(II) solution. As expected, CO gas or dissolved CO did not show the same type of reducing power and thus did not turn on fluorescence the same way.

### Does the DPHP-Cu(II) System Truly Have the Stated Selectivity Over Thiol Species?

**Figure 7:**
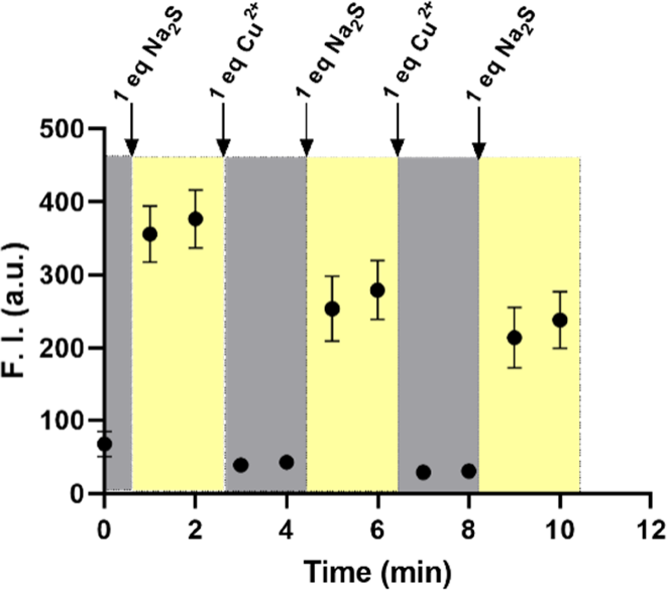
Effects of Na_2_S on the fluorescence of DPHP-Cu(II) solution (10 μM each) (*n* = 3, mean ± SD, λ_ex_ = 430 nm, λ_em_ = 582 nm, bandwidth = 3 nm).

In the original publication, the DPHP-Cu(II) solution was said to be stable and thiol species such as GSH, Cys, and H_2_S did not pose selectivity interference. Given the central role of Cu^2+^ in regulating the fluorescence of the proposed system and the known reactivity of thiol species with Cu^2+^, we sought to validate this claim through further experiments. It is well known that copper ion is very sensitive to sulfur, with the solubility constant *K*_sp_ for CuS being 6.3 × 10^–36^.^48^ Along this line, we were puzzled by the stated lack of response of the DPHP-Cu(II) system to various thiol species. To examine this, some widely used thiol compounds (Na_2_S, GSH, and Cys) were chosen to assess their ability to turn on the fluorescence of the DPHP-Cu(II) solution. We found that the DPHP-Cu(II) solution was very sensitive to the presence of these thiol species. 1 equiv of Na_2_S was able to fully turn on the fluorescence of the DPHP-Cu(II) solution quickly (Figure 7). In addition, the turn-on effect of Na_2_S was “recyclable,” at least to some degree. For example, the subsequent addition of another equivalent of Cu^2+^ after recovering the fluorescence by the sulfide was able to quench the fluorescence again. Such an on-and-off cycle can be repeated for at least three rounds (Figure 7), indicating the ability of Na_2_S to sequester Cu^2+^. During the recycling process, we noticed attenuated ability for the same amount of Na_2_S to turn on the fluorescence to the same level as it initially did. We are unsure of the reason(s) for this. One possibility is in the commercially available Na_2_S, which is in a hydrated form with undefined equivalents of water molecules. However, in our experiments, we only used the molecular formula of Na_2_S to do the theoretical calculation. This means that the actual amount of Na_2_S used is expected to be slightly less than 1 equiv in each round. This problem is expected to have cumulative effects, propagating the inadequacy of the fluorescence-restoring effects of Na_2_S. We also tested the influence of thiol compounds on the DPHP-Cu(II) solution at different concentrations (0.1, 1, and 5 mM) by using 0.1 mM CORM-3 as the positive control and found the full turn-on effects of these thiols species on the fluorescence of the DPHP-Cu(II) solution (Figure S3). Such results disagree with what was reported in the original publication and demonstrate selectivity issues over thiol species. We are not sure what the reason(s) might be for the observed discrepancy.

## Conclusions

In summary, we have reassessed the ability of the DPHP-Cu(II) solution to sense CO. First, spectroscopic and X-ray crystallographic characterizations confirmed the structural identity of DPHP. In our studies, the DPHP-Cu(II) solution: (1) senses CORM-3, not CO; (2) is sensitive to the presence of thiol species; (3) is sensitive to other reducing agents such as ascorbate, and (4) exhibits stability issues. We conclude that the DPHP-Cu(II) system is not a CO probe. If anything, it might qualify as a “CORM-3 probe,” a “sulfide probe,” or an “ascorbate probe,” but all without selectivity. At this point, the number of chemical feasibility issues identified led us to conclude that there is no need to attempt to duplicate the biology experiments in using this probe to image CO *in vitro* or *in vivo*. Further, copper is widely known to be toxic^49,50^ with applications in contraception (spermicidal)^51,52^ and sterilization (bactericidal).^50,53−55^ In bioimaging studies, the issue diffusion would mean that Cu^2+^ concentration would need to be high systemically to sense CORM-3. The presence of a large number of thiol species and reducing agents are expected to pose interference problems. The findings in this study further emphasize the need for using a pure CO source in assessing the sensing chemistry of a CO probe and the need to understand the fundamental chemical mechanism in developing CO probes. We hope that all future CO probe work will use more than a chemically reactive CORM as the sole CO source. This is actually a minimal requirement for developing a viable CO probe, especially considering the known chemical reactivity of the commonly used CORMs.^13−20,23,25−29,34^

## Supporting Information Available

Detailed data including supporting figures, experimental details, NMR spectra, and crystal data (PDF)
