# Supplementary material for: Sensing a CO-Releasing Molecule (CORM) Does Not Equate to Sensing CO: The Case of DPHP and CORM-3
Source: Anal Chem. 2023 Jun 1;95(23):9083–9. doi: 10.1021/acs.analchem.3c01495 (PMC10267888; doi:10.1021/acs.analchem.3c01495)
Supplement: Supplementary file 1 — ac3c01495_si_001.pdf [file ac3c01495_si_001.pdf]

Supporting Information

Sensing a CO-releasing Molecule (CORM) Does Not Equate to Sensing CO: The Case of DPHP and CORM-3

Dongning Liu, Xiaoxiao Yang, and Binghe Wang*

Department of Chemistry and Center for Diagnostics and Therapeutics, Georgia State University, Atlanta, Georgia 30303, USA. Email: wang@gsu.edu

Table of contents

Supplemental Figures S2
Experimental details S3
NMR spectra and Crystallographic data S5
Reference S8

Supplemental Figures

Scheme S1. Originally proposed mechanism for DPHP to sense CO.

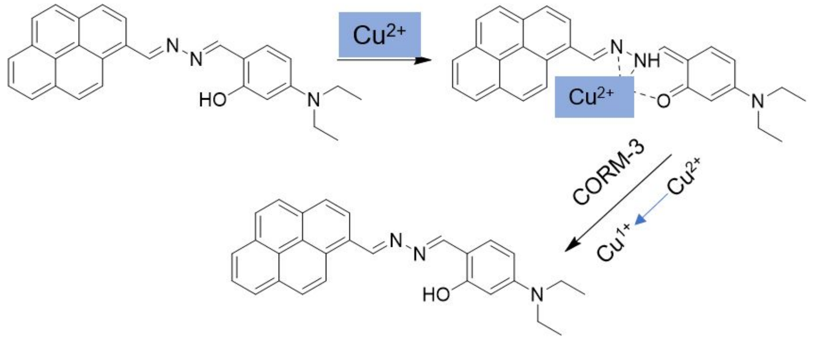

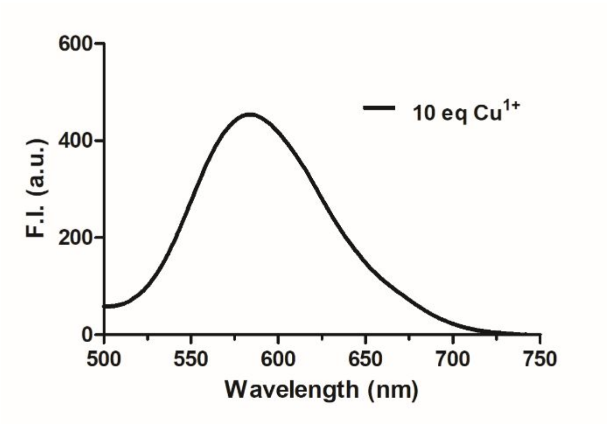

Figure S1. The effects of Cu^{1+} (10 eq) on the fluorescence of DPHP (10 μM) solution. (λ_{ex} = 430 nm, bandwidth = 3 nm)

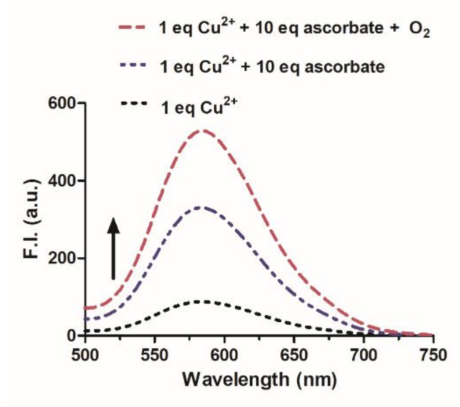

Figure S2. The effects of ascorbate on the fluorescence of DPHP-Cu(II) (10 μM) solution. The The solution was purged with oxygen for 5 mins. Sodium ascorbate was used in the experiments. (λ_{ex} = 430 nm, bandwidth = 3 nm)

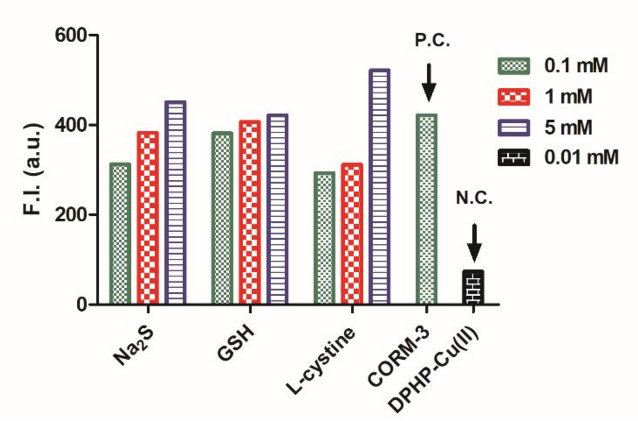

Figure S3. The effects of thiols species on the fluorescence of DPHP-Cu(II) (10 mM each) solution. 0.1 mM CORM-3 was used as a positive control and 0.01 mM DPHP-Cu(II) was used as a negative control. (λ_{ex} = 430 nm, λ_{em} = 582 nm, bandwidth = 3 nm)

Experimental details

Data for NMR are reported in terms of chemical shift (δ, ppm): Multiplicity (s = singlet, d =doublet, t = triplet, q = quartet, m = multiplet or unresolved, br = broad singlet, coupling constant(s) in Hz.).

Synthesis of the DPHP.

Scheme S2. The synthesis scheme of DPHP.

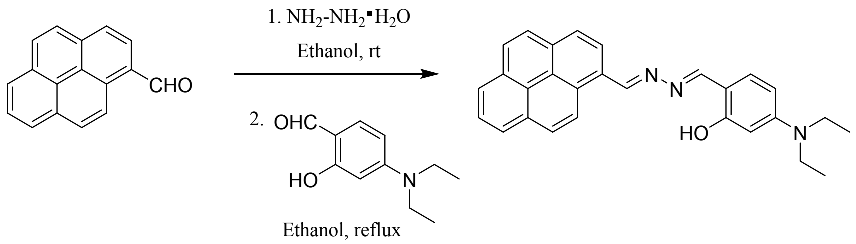

DPHP was synthesized by following a literature procedure.[1] Briefly, a hydrazine hydrate ethanol solution (1 mmol, 50 mg) was added to a pyrene-1-carbaldehyde (1 mmol, 232 mg) ethanol solution slowly. The reaction mixture was stirred at room temperature for 4 hours. Precipitate formation was observed. The precipitate was filtered with suction, rinsed with cold ethanol (50 mL), air-dried and then directly used without further purification. For the second step, the mixture of 4Å molecular sieve (0.5 g), 4-(diethylamino) salicylaldehyde (1 mmol, 199 mg) and the product from the first step was heated at reflux in ethanol for 8 h. After cooling down to room temperature, the solid precipitation and molecular sieve were collected with suction filtration; then the solid precipitation was dissolved in DCM, and the molecular sieve was removed by filtration. After

removing the DCM and recrystallizing in ethanol (30 mL), the final product, **DPHP**, was obtained as needle-shaped orange crystals. Yield: 201 mg (48%). HRMS: $[M+H]^+$ Calc. for $C_{28}H_{26}N_3O$: 420.2076; Found: 420.2094. ^1H NMR (400 MHz, DMSO-d_6): δ 11.81 (s, 1H), 9.72 (s, 1H), 9.14 (d, *J* = 9.4 Hz, 1H), 8.91 (s, 1H), 8.70 (d, *J* = 8.2 Hz, 1H), 8.45 – 8.20 (m, 6H), 8.14 (t, *J* = 7.6 Hz, 1H), 7.40 (d, *J* = 8.9 Hz, 1H), 6.37 (d, *J* = 8.8 Hz, 1H), 6.18 (d, *J* = 1.9 Hz, 1H), 3.42 (q, *J* = 6.9 Hz, 4H), 1.14 (t, *J* = 7.0 Hz, 6H). ^{13}C NMR (101 MHz, DMSO-d_6): δ 164.1, 161.4, 157.9, 151.5, 133.8, 132.5, 130.9, 130.2, 129.5, 128.8, 127.4, 126.9, 126.7, 126.2, 126.0, 126.0, 125.2, 124.2, 123.74, 123.4, 106.4, 104.3, 97.0, 44.0, 12.6.

Experiment procedures for spectroscopic studies

Fluorescence of DPHP and its response to Cu^{1+} addition. For Figure S1, CuCl was prepared as a 200 μM stock solution; as an example, 1.2 mg CuCl was weighed by microbalance, 60.6 mL of anhydrous and degassed CH_3CN was added under the protection of argon to get the stock solution. For the Cu^{1+} experiments, 735 μL of CuCl stock solution (200 μM), 750 μL deionized water, and 15 μL **DPHP** stock solution (1 mM) were added to a 1.5 mL cuvette to get the DPHP-Cu(II) solution. The spectrum was recorded under 37 °C.

Effects of ascorbate on the fluorescence of DPHP-Cu(II) solution. Sodium ascorbate was prepared as 10 mM stock solution. For Figure S2, 15 μL (10 eq) of sodium ascorbate stock solution (10 mM) was added to the DPHP-Cu(II) solutions (both 10 μM) separately and mixed by pipetting and releasing. Oxygen was bubbling into this solution for 5 mins. The spectrums were recorded under 37 °C.

The effects of thiol species on the fluorescence of DPHP. For Figure S4**,** thiol species was prepared as a 10-mM stock solution. For the effects of thiol species on the fluorescence of **DPHP**, 1.5 μL (1 eq) of the thiol species stock solution (10 mM) was added to the DPHP-Cu(II) solutions (10 μM each) and mixed by pipetting and releasing. After the reading became stable, 1.5 μL (1 eq) of Cu^{2+} stock solution was added and mixed by pipetting and releasing. These steps were repeated for another 2 times to complete this experiment. Data were recorded under 37 °C. For Figure S5, 10-mM thiol stock solutions were prepared in deionized water. For example, 735 μL of CH_3CN, 15 μL **DPHP** stock solution (1 mM), 1.5 μL Cu^{2+} stock solution (10 mM), and 750 μL thiol stock solution(10 mM), and were added to a 1.5 mL cuvette to make 5 mM thiols in DPHP-Cu(II) solution. Similar procedure for 0.1 mM and 1 mM groups. The data were recorded under 37 °C.

NMR spectra and Crystallographic data

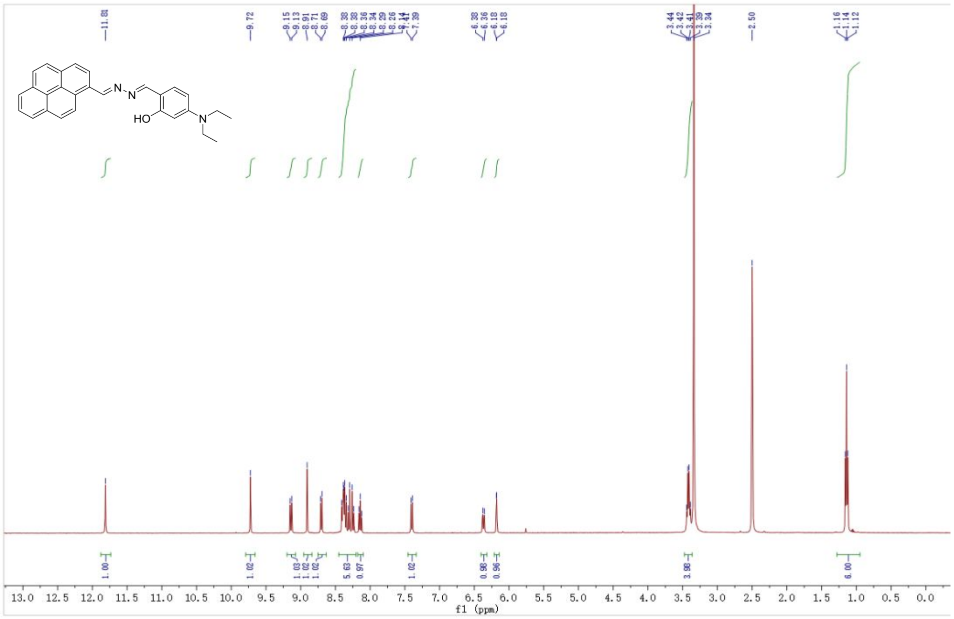

Figure S4. ^{1}H NMR spectrum of DPHP in DMSO-d_6.

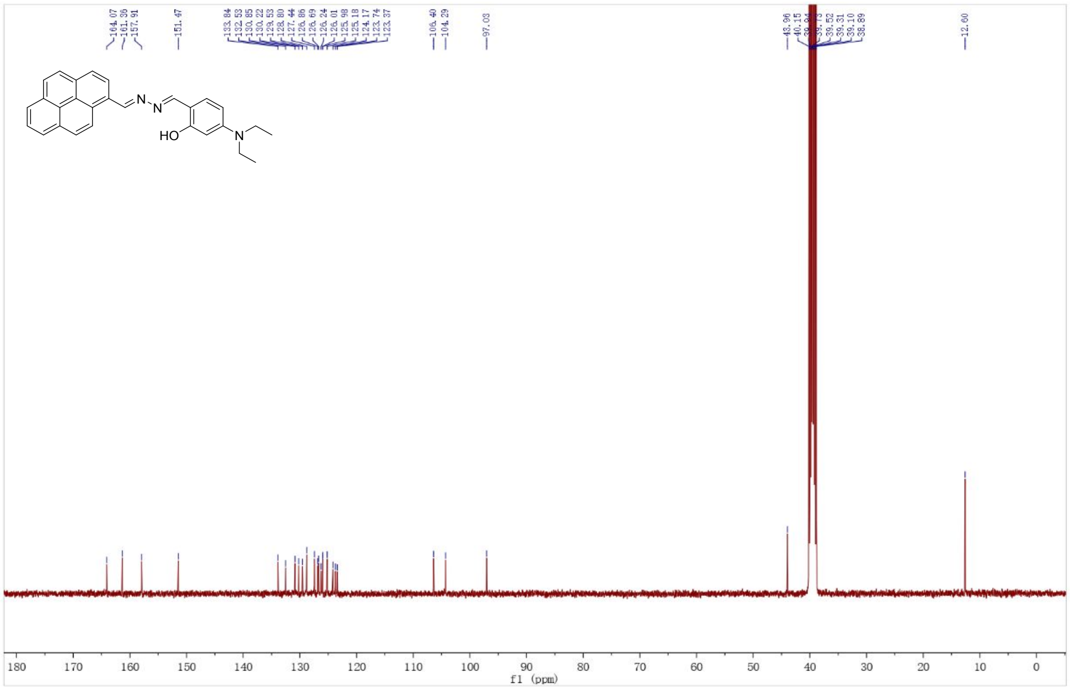

Figure S5. ^{13}C NMR spectrum of DPHP in DMSO-d_6.

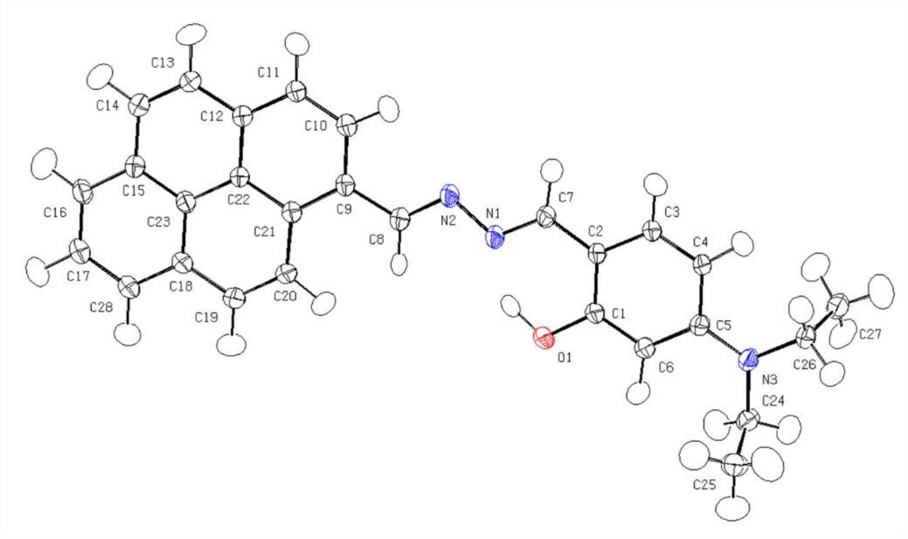

Figure S6. DPHP X-ray crystallographic ellipsoid plot (at the 50% probability level, CCDC 2244511 contains the supplementary crystallographic data).

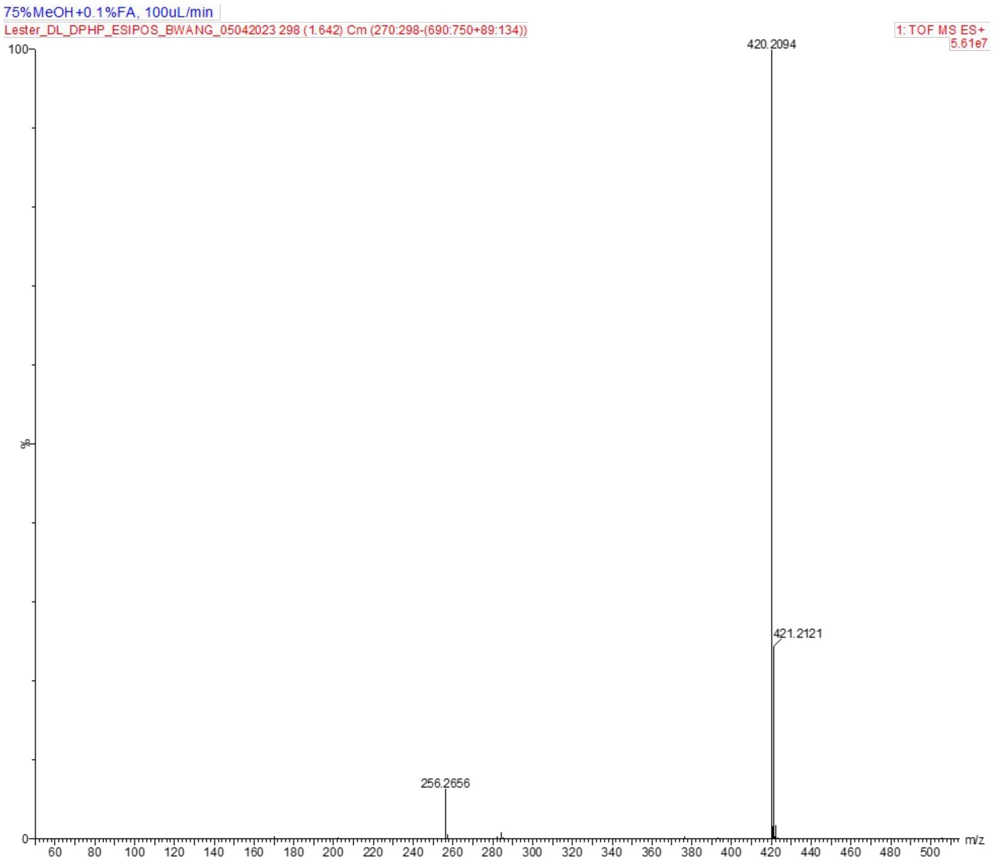

Figure S7. HRMS spectrum (ESI+) of DPHP.

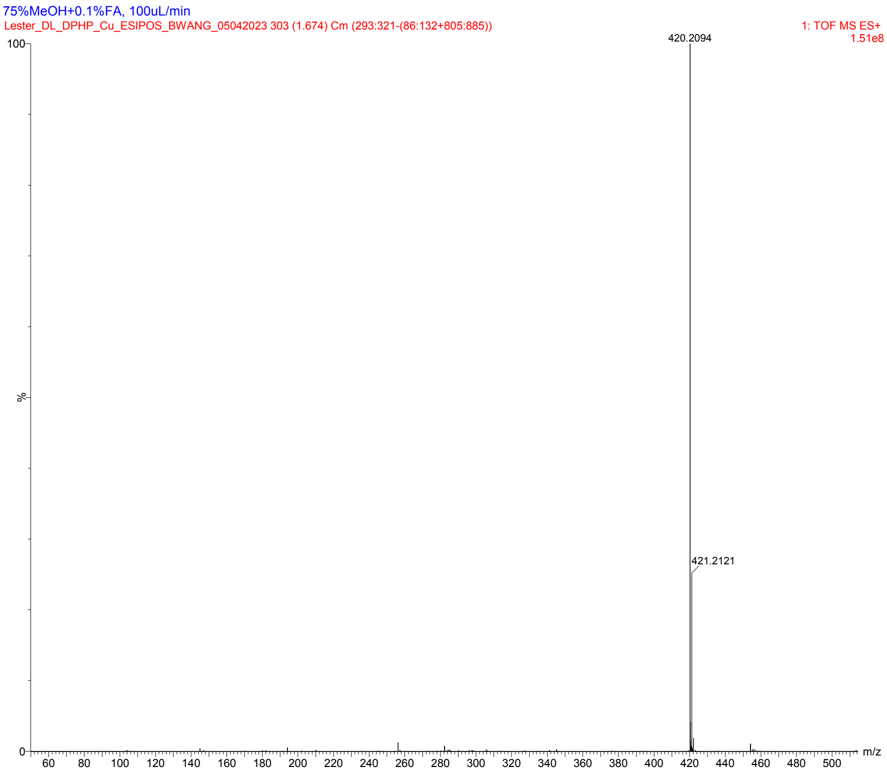

Figure S8. HRMS spectrum (ESI+) of DPHP-Cu(II)

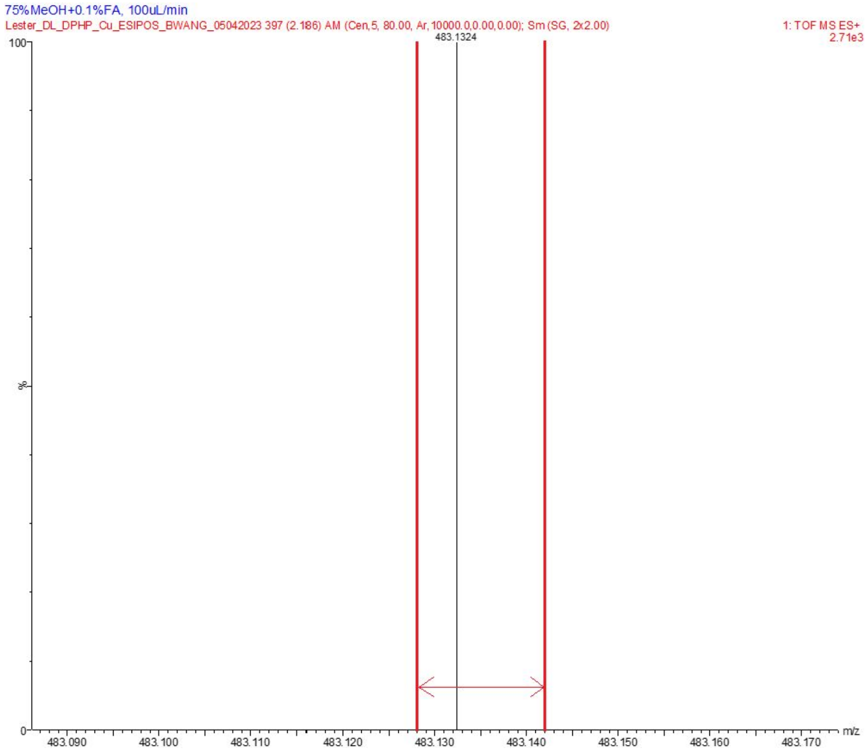

Figure S9. HRMS spectrum (ESI+) of the DPHP-Cu(II) solution amplified by 50,000-fold in the narrow region of 483.090 to 483.170 (the region between the red marks is the zoomed-in range used in the original publication[1]).

Table S1. Crystallographic data of DPHP

Parameter	Data
Formula	$C_{28}H_{25}N_3O$
$D_{calc.}$/ g cm^{-3}	1.299
μ/mm^{-1}	0.626
Formula Weight	419.530
Color	orange
Shape	needle-shaped
Size/mm^3	0.33×0.06×0.03
T/K	100.00(10)
Crystal System	triclinic
Space Group	P-1
a/Å	8.3093(2)
b/Å	12.2273(3)
c/Å	22.3350(5)
α/°	101.605(2)
β/°	96.467(2)
γ/°	102.128(2)
V/Å^3	2144.63(9)
Z	4
Z'	2
Wavelength/Å	1.54184
Radiation type	Cu K_α
Θ_{min}/°	3.80
Θ_{max}/°	73.16
Measured Refl's.	27319
Indep't Refl's	8110
Refl's I≥2 σ(I)	5659
R_{int}	0.0593
Parameters	1027
Restraints	978
Largest Peak	0.3277
Deepest Hole	-0.2652
GooF	1.0311
wR_2 (all data)	0.1121
wR_2	0.1006
R_1 (all data)	0.0688
R_1	0.0435

Reference

(1) Bai, C.; Zhang, J.; Qin, Y.; Meng, Q.; Yao, J.; Huang, H.; Wei, B.; Li, R.; Zhang, L.; Miao, H.; Qu, C.; Qiao, R. Strategy for Detecting Carbon Monoxide: Cu2+-Assisted Fluorescent Probe and Its Applications in Biological Imaging. *Anal. Chem.* **2022**, *94* (32), 11298–11306.
